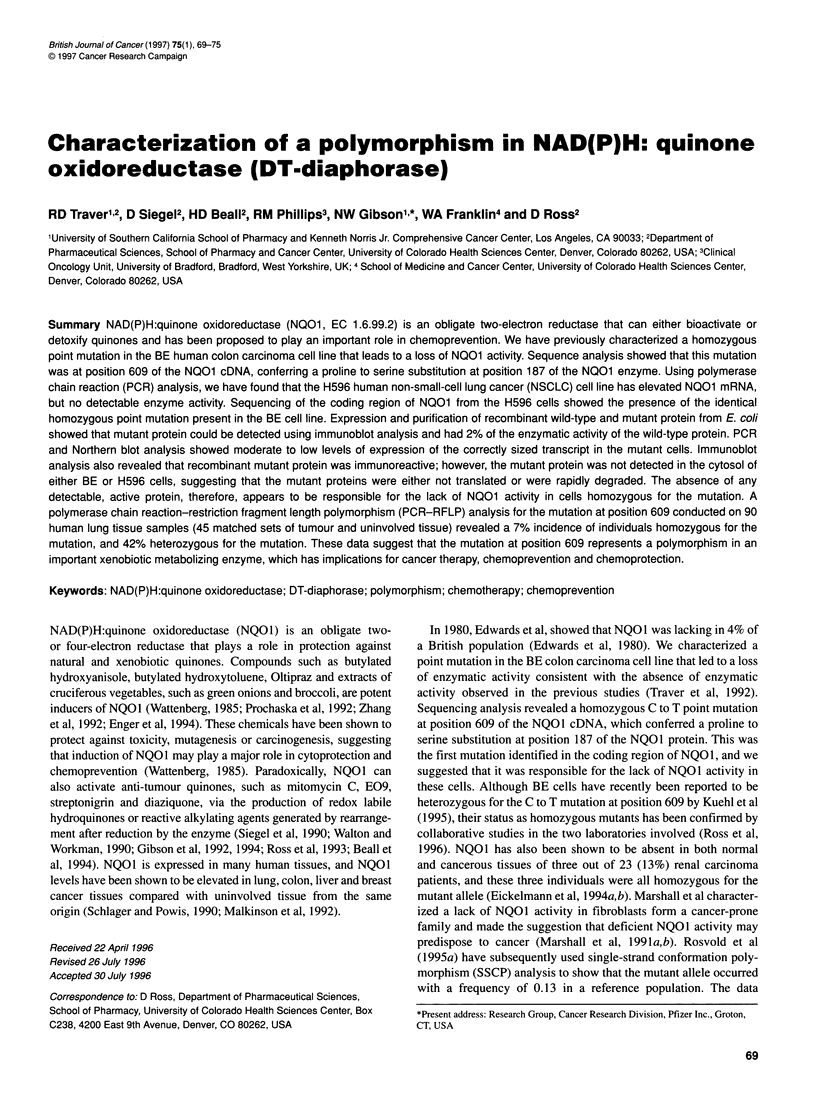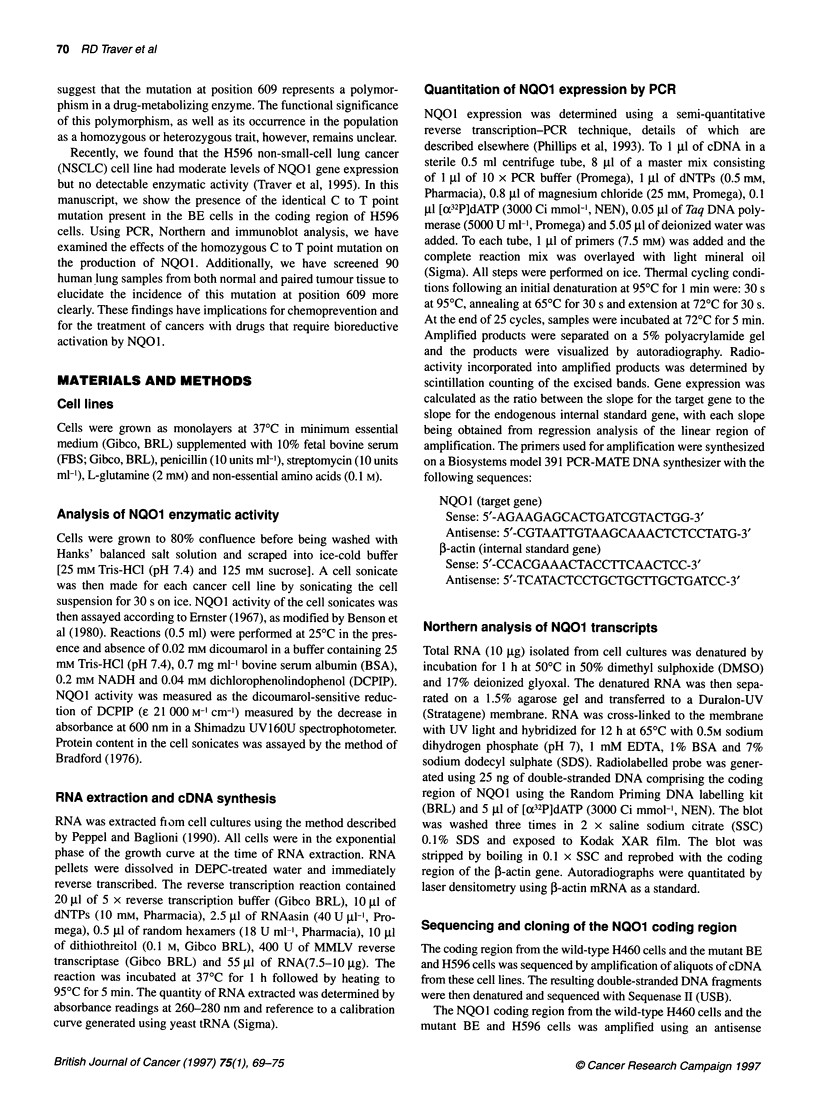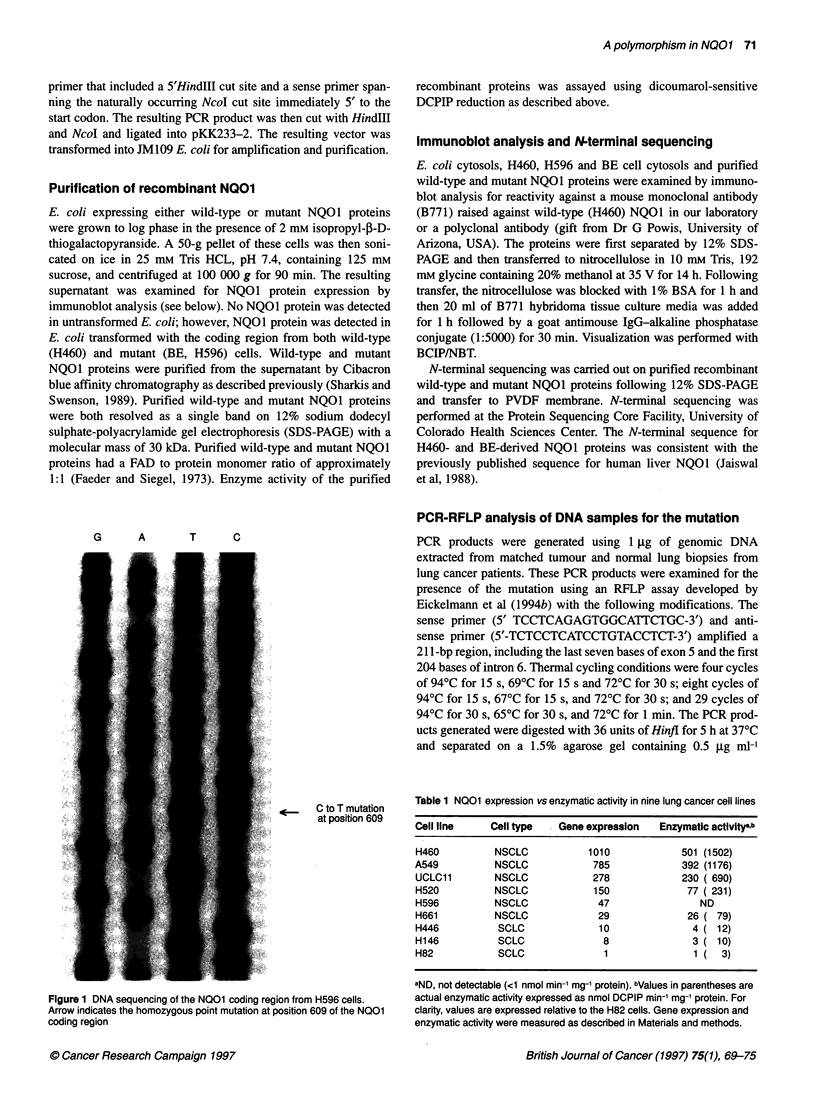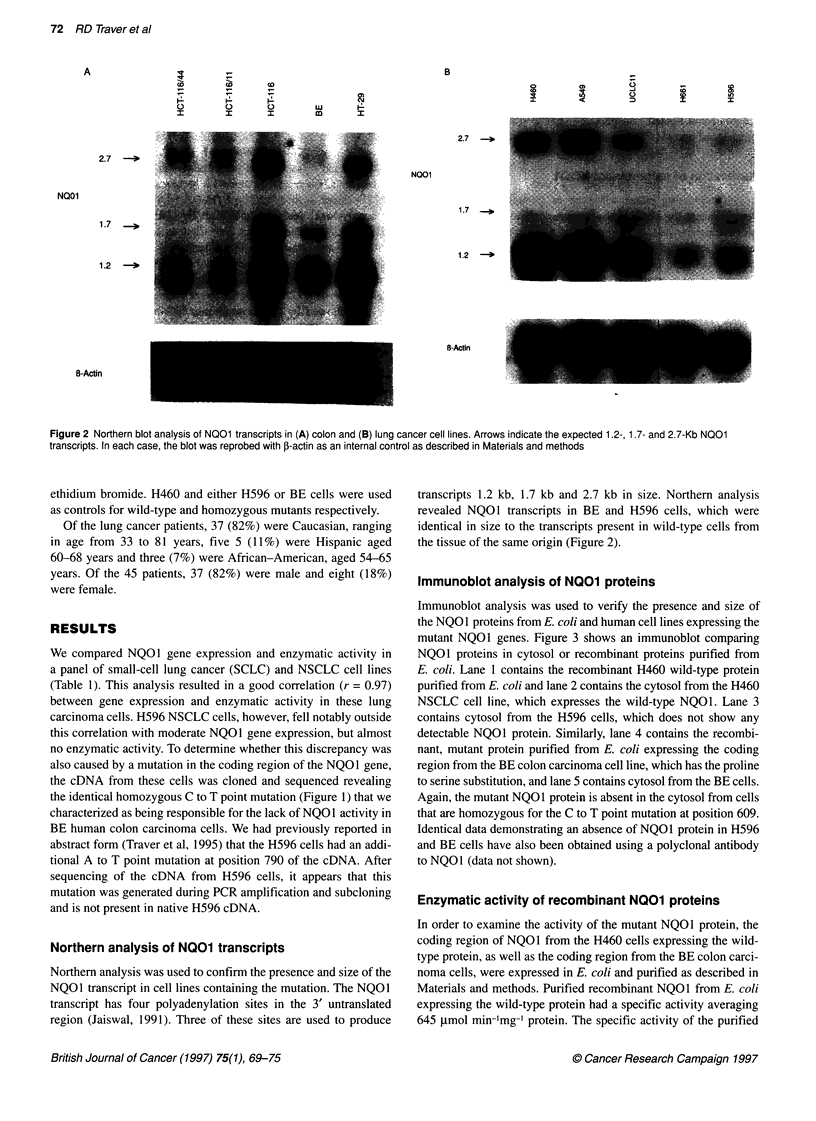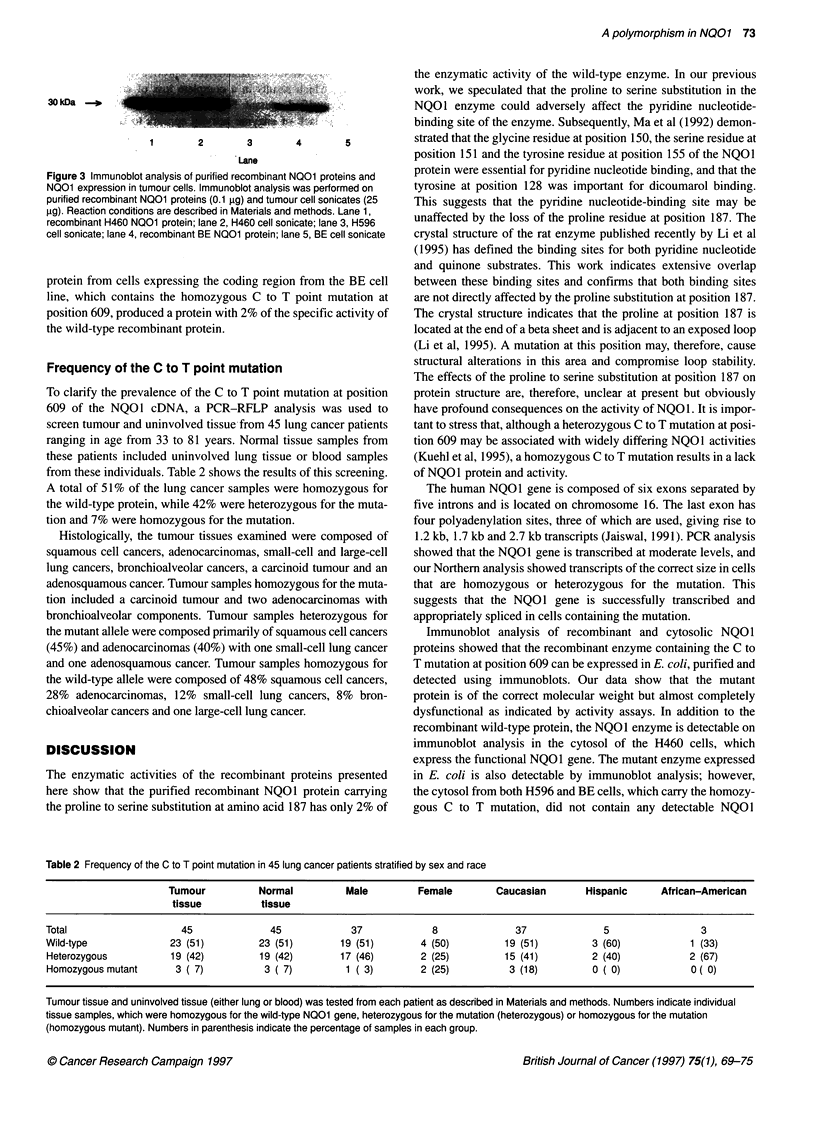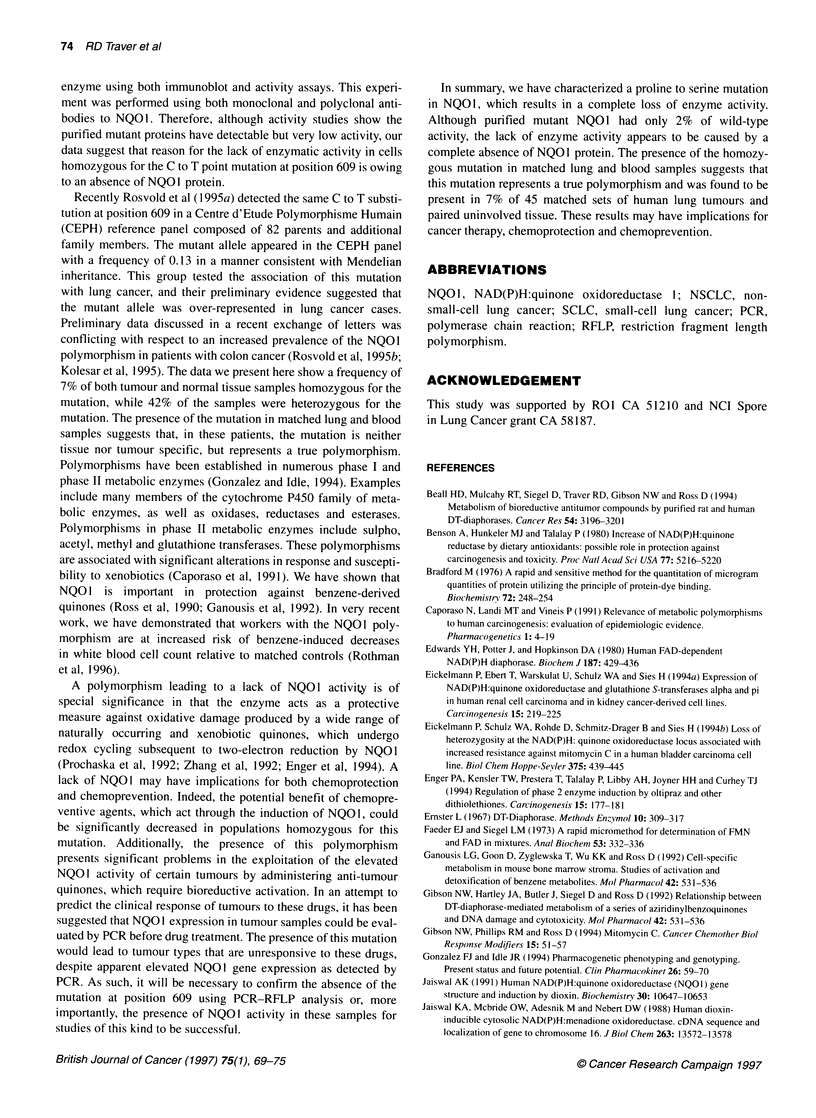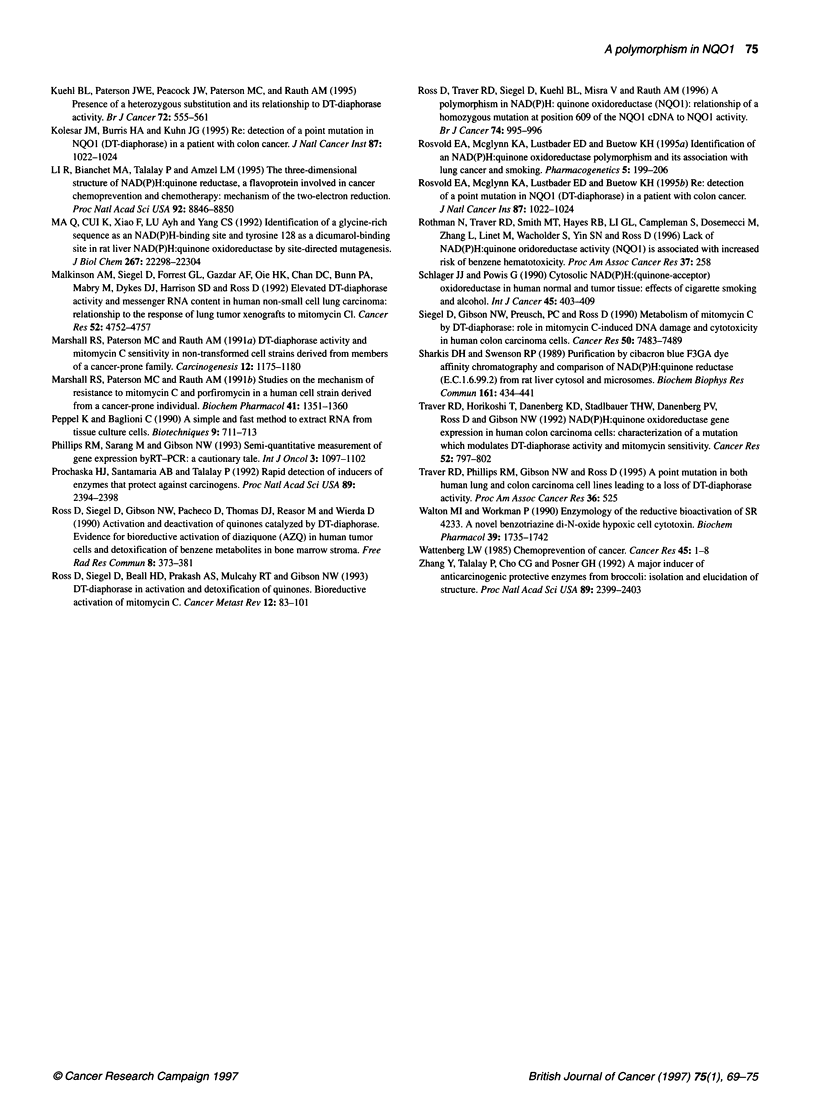# Characterization of a polymorphism in NAD(P)H: quinone oxidoreductase (DT-diaphorase).

**DOI:** 10.1038/bjc.1997.11

**Published:** 1997

**Authors:** R. D. Traver, D. Siegel, H. D. Beall, R. M. Phillips, N. W. Gibson, W. A. Franklin, D. Ross

**Affiliations:** University of Southern California School of Pharmacy and Kenneth Norris Jr. Comprehensive Cancer Center, Los Angeles 90033, USA.

## Abstract

**Images:**


British Journal of Cancer (1997) 75(1), 69–75
© 1997 Cancer Research Campaign

### Characterization of a polymorphism in NAD(P)H: quinone oxidoreductase (DT-diaphorase)

**RD Traver[1,2], D Siegel[2], HD Beall[2], RM Phillips[3], NW Gibson[1,*], WA Franklin[4] and D Ross[2]**

[1]University of Southern California School of Pharmacy and Kenneth Norris Jr. Comprehensive Cancer Center, Los Angeles, CA 90033; [2]Department of Pharmaceutical Sciences, School of Pharmacy and Cancer Center, University of Colorado Health Sciences Center, Denver, Colorado 80262, USA; [3]Clinical Oncology Unit, University of Bradford, Bradford, West Yorkshire, UK; [4] School of Medicine and Cancer Center, University of Colorado Health Sciences Center, Denver, Colorado 80262, USA

**Summary** NAD(P)H:quinone oxidoreductase (NQO1, EC 1.6.99.2) is an obligate two-electron reductase that can either bioactivate or detoxify quinones and has been proposed to play an important role in chemoprevention. We have previously characterized a homozygous point mutation in the BE human colon carcinoma cell line that leads to a loss of NQO1 activity. Sequence analysis showed that this mutation was at position 609 of the NQO1 cDNA, conferring a proline to serine substitution at position 187 of the NQO1 enzyme. Using polymerase chain reaction (PCR) analysis, we have found that the H596 human non-small-cell lung cancer (NSCLC) cell line has elevated NQO1 mRNA, but no detectable enzyme activity. Sequencing of the coding region of NQO1 from the H596 cells showed the presence of the identical homozygous point mutation present in the BE cell line. Expression and purification of recombinant wild-type and mutant protein from *E. coli* showed that mutant protein could be detected using immunoblot analysis and had 2% of the enzymatic activity of the wild-type protein. PCR and Northern blot analysis showed moderate to low levels of expression of the correctly sized transcript in the mutant cells. Immunoblot analysis also revealed that recombinant mutant protein was immunoreactive; however, the mutant protein was not detected in the cytosol of either BE or H596 cells, suggesting that the mutant proteins were either not translated or were rapidly degraded. The absence of any detectable, active protein, therefore, appears to be responsible for the lack of NQO1 activity in cells homozygous for the mutation. A polymerase chain reaction–restriction fragment length polymorphism (PCR–RFLP) analysis for the mutation at position 609 conducted on 90 human lung tissue samples (45 matched sets of tumour and uninvolved tissue) revealed a 7% incidence of individuals homozygous for the mutation, and 42% heterozygous for the mutation. These data suggest that the mutation at position 609 represents a polymorphism in an important xenobiotic metabolizing enzyme, which has implications for cancer therapy, chemoprevention and chemoprotection.

**Keywords**: NAD(P)H:quinone oxidoreductase; DT-diaphorase; polymorphism; chemotherapy; chemoprevention

NAD(P)H:quinone oxidoreductase (NQO1) is an obligate two- or four-electron reductase that plays a role in protection against natural and xenobiotic quinones. Compounds such as butylated hydroxyanisole, butylated hydroxytoluene, Oltipraz and extracts of cruciferous vegetables, such as green onions and broccoli, are potent inducers of NQO1 (Wattenberg, 1985; Prochaska et al, 1992; Zhang et al, 1992; Enger et al, 1994). These chemicals have been shown to protect against toxicity, mutagenesis or carcinogenesis, suggesting that induction of NQO1 may play a major role in cytoprotection and chemoprevention (Wattenberg, 1985). Paradoxically, NQO1 can also activate anti-tumour quinones, such as mitomycin C, EO9, streptonigrin and diaziquone, via the production of redox labile hydroquinones or reactive alkylating agents generated by rearrangement after reduction by the enzyme (Siegel et al, 1990; Walton and Workman, 1990; Gibson et al, 1992, 1994; Ross et al, 1993; Beall et al, 1994). NQO1 is expressed in many human tissues, and NQO1 levels have been shown to be elevated in lung, colon, liver and breast cancer tissues compared with uninvolved tissue from the same origin (Schlager and Powis, 1990; Malkinson et al, 1992).

*Received 22 April 1996*
*Revised 26 July 1996*
*Accepted 30 July 1996*

*Correspondence to:* D Ross, Department of Pharmaceutical Sciences, School of Pharmacy, University of Colorado Health Sciences Center, Box C238, 4200 East 9th Avenue, Denver, CO 80262, USA

In 1980, Edwards et al, showed that NQO1 was lacking in 4% of a British population (Edwards et al, 1980). We characterized a point mutation in the BE colon carcinoma cell line that led to a loss of enzymatic activity consistent with the absence of enzymatic activity observed in the previous studies (Traver et al, 1992). Sequencing analysis revealed a homozygous C to T point mutation at position 609 of the NQO1 cDNA, which conferred a proline to serine substitution at position 187 of the NQO1 protein. This was the first mutation identified in the coding region of NQO1, and we suggested that it was responsible for the lack of NQO1 activity in these cells. Although BE cells have recently been reported to be heterozygous for the C to T mutation at position 609 by Kuehl et al (1995), their status as homozygous mutants has been confirmed by collaborative studies in the two laboratories involved (Ross et al, 1996). NQO1 has also been shown to be absent in both normal and cancerous tissues of three out of 23 (13%) renal carcinoma patients, and these three individuals were all homozygous for the mutant allele (Eickelmann et al, 1994*a*,*b*). Marshall et al characterized a lack of NQO1 activity in fibroblasts form a cancer-prone family and made the suggestion that deficient NQO1 activity may predispose to cancer (Marshall et al, 1991*a*,*b*). Rosvold et al (1995*a*) have subsequently used single-strand conformation polymorphism (SSCP) analysis to show that the mutant allele occurred with a frequency of 0.13 in a reference population. The data

*Present address: Research Group, Cancer Research Division, Pfizer Inc., Groton, CT, USA

suggest that the mutation at position 609 represents a polymorphism in a drug-metabolizing enzyme. The functional significance of this polymorphism, as well as its occurrence in the population as a homozygous or heterozygous trait, however, remains unclear.

Recently, we found that the H596 non-small-cell lung cancer (NSCLC) cell line had moderate levels of NQO1 gene expression but no detectable enzymatic activity (Traver et al, 1995). In this manuscript, we show the presence of the identical C to T point mutation present in the BE cells in the coding region of H596 cells. Using PCR, Northern and immunoblot analysis, we have examined the effects of the homozygous C to T point mutation on the production of NQO1. Additionally, we have screened 90 human lung samples from both normal and paired tumour tissue to elucidate the incidence of this mutation at position 609 more clearly. These findings have implications for chemoprevention and for the treatment of cancers with drugs that require bioreductive activation by NQO1.

### MATERIALS AND METHODS

### Cell lines

Cells were grown as monolayers at 37°C in minimum essential medium (Gibco, BRL) supplemented with 10% fetal bovine serum (FBS; Gibco, BRL), penicillin (10 units $ml^{-1}$), streptomycin (10 units $ml^{-1}$), L-glutamine (2 mM) and non-essential amino acids (0.1 M).

### Analysis of NQO1 enzymatic activity

Cells were grown to 80% confluence before being washed with Hanks' balanced salt solution and scraped into ice-cold buffer [25 mM Tris-HCl (pH 7.4) and 125 mM sucrose]. A cell sonicate was then made for each cancer cell line by sonicating the cell suspension for 30 s on ice. NQO1 activity of the cell sonicates was then assayed according to Ernster (1967), as modified by Benson et al (1980). Reactions (0.5 ml) were performed at 25°C in the presence and absence of 0.02 mM dicoumarol in a buffer containing 25 mM Tris-HCl (pH 7.4), 0.7 mg $ml^{-1}$ bovine serum albumin (BSA), 0.2 mM NADH and 0.04 mM dichlorophenolindophenol (DCPIP). NQO1 activity was measured as the dicoumarol-sensitive reduction of DCPIP ($\varepsilon$ 21 000 $M^{-1}$ $cm^{-1}$) measured by the decrease in absorbance at 600 nm in a Shimadzu UV160U spectrophotometer. Protein content in the cell sonicates was assayed by the method of Bradford (1976).

### RNA extraction and cDNA synthesis

RNA was extracted fıom cell cultures using the method described by Peppel and Baglioni (1990). All cells were in the exponential phase of the growth curve at the time of RNA extraction. RNA pellets were dissolved in DEPC-treated water and immediately reverse transcribed. The reverse transcription reaction contained 20 μl of 5 × reverse transcription buffer (Gibco BRL), 10 μl of dNTPs (10 mM, Pharmacia), 2.5 μl of RNAasin (40 U $\mu l^{-1}$, Promega), 0.5 μl of random hexamers (18 U $ml^{-1}$, Pharmacia), 10 μl of dithiothreitol (0.1 M, Gibco BRL), 400 U of MMLV reverse transcriptase (Gibco BRL) and 55 μl of RNA(7.5–10 μg). The reaction was incubated at 37°C for 1 h followed by heating to 95°C for 5 min. The quantity of RNA extracted was determined by absorbance readings at 260–280 nm and reference to a calibration curve generated using yeast tRNA (Sigma).

### Quantitation of NQO1 expression by PCR

NQO1 expression was determined using a semi-quantitative reverse transcription–PCR technique, details of which are described elsewhere (Phillips et al, 1993). To 1 μl of cDNA in a sterile 0.5 ml centrifuge tube, 8 μl of a master mix consisting of 1 μl of 10 × PCR buffer (Promega), 1 μl of dNTPs (0.5 mM, Pharmacia), 0.8 μl of magnesium chloride (25 mM, Promega), 0.1 μl [$\alpha^{32}P$]dATP (3000 Ci $mmol^{-1}$, NEN), 0.05 μl of *Taq* DNA polymerase (5000 U $ml^{-1}$, Promega) and 5.05 μl of deionized water was added. To each tube, 1 μl of primers (7.5 mM) was added and the complete reaction mix was overlayed with light mineral oil (Sigma). All steps were performed on ice. Thermal cycling conditions following an initial denaturation at 95°C for 1 min were: 30 s at 95°C, annealing at 65°C for 30 s and extension at 72°C for 30 s. At the end of 25 cycles, samples were incubated at 72°C for 5 min. Amplified products were separated on a 5% polyacrylamide gel and the products were visualized by autoradiography. Radioactivity incorporated into amplified products was determined by scintillation counting of the excised bands. Gene expression was calculated as the ratio between the slope for the target gene to the slope for the endogenous internal standard gene, with each slope being obtained from regression analysis of the linear region of amplification. The primers used for amplification were synthesized on a Biosystems model 391 PCR-MATE DNA synthesizer with the following sequences:

NQO1 (target gene)
Sense: 5′-AGAAGAGCACTGATCGTACTGG-3′
Antisense: 5′-CGTAATTGTAAGCAAACTCTCCTATG-3′
β-actin (internal standard gene)
Sense: 5′-CCACGAAACTACCTTCAACTCC-3′
Antisense: 5′-TCATACTCCTGCTGCTTGCTGATCC-3′

### Northern analysis of NQO1 transcripts

Total RNA (10 μg) isolated from cell cultures was denatured by incubation for 1 h at 50°C in 50% dimethyl sulphoxide (DMSO) and 17% deionized glyoxal. The denatured RNA was then separated on a 1.5% agarose gel and transferred to a Duralon-UV (Stratagene) membrane. RNA was cross-linked to the membrane with UV light and hybridized for 12 h at 65°C with 0.5M sodium dihydrogen phosphate (pH 7), 1 mM EDTA, 1% BSA and 7% sodium dodecyl sulphate (SDS). Radiolabelled probe was generated using 25 ng of double-stranded DNA comprising the coding region of NQO1 using the Random Priming DNA labelling kit (BRL) and 5 μl of [$\alpha^{32}P$]dATP (3000 Ci $mmol^{-1}$, NEN). The blot was washed three times in 2 × saline sodium citrate (SSC) 0.1% SDS and exposed to Kodak XAR film. The blot was stripped by boiling in 0.1 × SSC and reprobed with the coding region of the β-actin gene. Autoradiographs were quantitated by laser densitometry using β-actin mRNA as a standard.

### Sequencing and cloning of the NQO1 coding region

The coding region from the wild-type H460 cells and the mutant BE and H596 cells was sequenced by amplification of aliquots of cDNA from these cell lines. The resulting double-stranded DNA fragments were then denatured and sequenced with Sequenase II (USB).

The NQO1 coding region from the wild-type H460 cells and the mutant BE and H596 cells was amplified using an antisense

© Cancer Research Campaign 1997

primer that included a 5′*Hin*dIII cut site and a sense primer spanning the naturally occurring *Nco*I cut site immediately 5′ to the start codon. The resulting PCR product was then cut with *Hin*dIII and *Nco*I and ligated into pKK233–2. The resulting vector was transformed into JM109 *E. coli* for amplification and purification.

### Purification of recombinant NQO1

*E. coli* expressing either wild-type or mutant NQO1 proteins were grown to log phase in the presence of 2 mM isopropyl-β-D-thiogalactopyranside. A 50-g pellet of these cells was then sonicated on ice in 25 mM Tris HCL, pH 7.4, containing 125 mM sucrose, and centrifuged at 100 000 *g* for 90 min. The resulting supernatant was examined for NQO1 protein expression by immunoblot analysis (see below). No NQO1 protein was detected in untransformed *E. coli*; however, NQO1 protein was detected in *E. coli* transformed with the coding region from both wild-type (H460) and mutant (BE, H596) cells. Wild-type and mutant NQO1 proteins were purified from the supernatant by Cibacron blue affinity chromatography as described previously (Sharkis and Swenson, 1989). Purified wild-type and mutant NQO1 proteins were both resolved as a single band on 12% sodium dodecyl sulphate-polyacrylamide gel electrophoresis (SDS-PAGE) with a molecular mass of 30 kDa. Purified wild-type and mutant NQO1 proteins had a FAD to protein monomer ratio of approximately 1:1 (Faeder and Siegel, 1973). Enzyme activity of the purified recombinant proteins was assayed using dicoumarol-sensitive DCPIP reduction as described above.

**Figure 1** DNA sequencing of the NQO1 coding region from H596 cells. Arrow indicates the homozygous point mutation at position 609 of the NQO1 coding region

### Immunoblot analysis and *N*-terminal sequencing

*E. coli* cytosols, H460, H596 and BE cell cytosols and purified wild-type and mutant NQO1 proteins were examined by immunoblot analysis for reactivity against a mouse monoclonal antibody (B771) raised against wild-type (H460) NQO1 in our laboratory or a polyclonal antibody (gift from Dr G Powis, University of Arizona, USA). The proteins were first separated by 12% SDS-PAGE and then transferred to nitrocellulose in 10 mM Tris, 192 mM glycine containing 20% methanol at 35 V for 14 h. Following transfer, the nitrocellulose was blocked with 1% BSA for 1 h and then 20 ml of B771 hybridoma tissue culture media was added for 1 h followed by a goat antimouse IgG–alkaline phosphatase conjugate (1:5000) for 30 min. Visualization was performed with BCIP/NBT.

*N*-terminal sequencing was carried out on purified recombinant wild-type and mutant NQO1 proteins following 12% SDS-PAGE and transfer to PVDF membrane. *N*-terminal sequencing was performed at the Protein Sequencing Core Facility, University of Colorado Health Sciences Center. The *N*-terminal sequence for H460- and BE-derived NQO1 proteins was consistent with the previously published sequence for human liver NQO1 (Jaiswal et al, 1988).

### PCR-RFLP analysis of DNA samples for the mutation

PCR products were generated using 1 μg of genomic DNA extracted from matched tumour and normal lung biopsies from lung cancer patients. These PCR products were examined for the presence of the mutation using an RFLP assay developed by Eickelmann et al (1994*b*) with the following modifications. The sense primer (5′ TCCTCAGAGTGGCATTCTGC-3′) and antisense primer (5′-TCTCCTCATCCTGTACCTCT-3′) amplified a 211-bp region, including the last seven bases of exon 5 and the first 204 bases of intron 6. Thermal cycling conditions were four cycles of 94°C for 15 s, 69°C for 15 s and 72°C for 30 s; eight cycles of 94°C for 15 s, 67°C for 15 s, and 72°C for 30 s; and 29 cycles of 94°C for 30 s, 65°C for 30 s, and 72°C for 1 min. The PCR products generated were digested with 36 units of *Hinf*I for 5 h at 37°C and separated on a 1.5% agarose gel containing 0.5 μg $ml^{-1}$

**Table 1** NQO1 expression *vs* enzymatic activity in nine lung cancer cell lines

| Cell line | Cell type | Gene expression | Enzymatic activity[a,b] |
|---|---|---|---|
| H460 | NSCLC | 1010 | 501 (1502) |
| A549 | NSCLC | 785 | 392 (1176) |
| UCLC11 | NSCLC | 278 | 230 ( 690) |
| H520 | NSCLC | 150 | 77 ( 231) |
| H596 | NSCLC | 47 | ND |
| H661 | NSCLC | 29 | 26 ( 79) |
| H446 | SCLC | 10 | 4 ( 12) |
| H146 | SCLC | 8 | 3 ( 10) |
| H82 | SCLC | 1 | 1 ( 3) |

[a]ND, not detectable (<1 nmol $min^{-1}$ $mg^{-1}$ protein). [b]Values in parentheses are actual enzymatic activity expressed as nmol DCPIP $min^{-1}$ $mg^{-1}$ protein. For clarity, values are expressed relative to the H82 cells. Gene expression and enzymatic activity were measured as described in Materials and methods.

© Cancer Research Campaign 1997

**Figure 2** Northern blot analysis of NQO1 transcripts in (**A**) colon and (**B**) lung cancer cell lines. Arrows indicate the expected 1.2-, 1.7- and 2.7-Kb NQO1 transcripts. In each case, the blot was reprobed with β-actin as an internal control as described in Materials and methods ethidium bromide. H460 and either H596 or BE cells were used as controls for wild-type and homozygous mutants respectively.

Of the lung cancer patients, 37 (82%) were Caucasian, ranging in age from 33 to 81 years, five 5 (11%) were Hispanic aged 60–68 years and three (7%) were African–American, aged 54–65 years. Of the 45 patients, 37 (82%) were male and eight (18%) were female.

### RESULTS

We compared NQO1 gene expression and enzymatic activity in a panel of small-cell lung cancer (SCLC) and NSCLC cell lines (Table 1). This analysis resulted in a good correlation ($r = 0.97$) between gene expression and enzymatic activity in these lung carcinoma cells. H596 NSCLC cells, however, fell notably outside this correlation with moderate NQO1 gene expression, but almost no enzymatic activity. To determine whether this discrepancy was also caused by a mutation in the coding region of the NQO1 gene, the cDNA from these cells was cloned and sequenced revealing the identical homozygous C to T point mutation (Figure 1) that we characterized as being responsible for the lack of NQO1 activity in BE human colon carcinoma cells. We had previously reported in abstract form (Traver et al, 1995) that the H596 cells had an additional A to T point mutation at position 790 of the cDNA. After sequencing of the cDNA from H596 cells, it appears that this mutation was generated during PCR amplification and subcloning and is not present in native H596 cDNA.

### Northern analysis of NQO1 transcripts

Northern analysis was used to confirm the presence and size of the NQO1 transcript in cell lines containing the mutation. The NQO1 transcript has four polyadenylation sites in the 3′ untranslated region (Jaiswal, 1991). Three of these sites are used to produce transcripts 1.2 kb, 1.7 kb and 2.7 kb in size. Northern analysis revealed NQO1 transcripts in BE and H596 cells, which were identical in size to the transcripts present in wild-type cells from the tissue of the same origin (Figure 2).

### Immunoblot analysis of NQO1 proteins

Immunoblot analysis was used to verify the presence and size of the NQO1 proteins from *E. coli* and human cell lines expressing the mutant NQO1 genes. Figure 3 shows an immunoblot comparing NQO1 proteins in cytosol or recombinant proteins purified from *E. coli*. Lane 1 contains the recombinant H460 wild-type protein purified from *E. coli* and lane 2 contains the cytosol from the H460 NSCLC cell line, which expresses the wild-type NQO1. Lane 3 contains cytosol from the H596 cells, which does not show any detectable NQO1 protein. Similarly, lane 4 contains the recombinant, mutant protein purified from *E. coli* expressing the coding region from the BE colon carcinoma cell line, which has the proline to serine substitution, and lane 5 contains cytosol from the BE cells. Again, the mutant NQO1 protein is absent in the cytosol from cells that are homozygous for the C to T point mutation at position 609. Identical data demonstrating an absence of NQO1 protein in H596 and BE cells have also been obtained using a polyclonal antibody to NQO1 (data not shown).

### Enzymatic activity of recombinant NQO1 proteins

In order to examine the activity of the mutant NQO1 protein, the coding region of NQO1 from the H460 cells expressing the wild-type protein, as well as the coding region from the BE colon carcinoma cells, were expressed in *E. coli* and purified as described in Materials and methods. Purified recombinant NQO1 from *E. coli* expressing the wild-type protein had a specific activity averaging 645 μmol $min^{-1}mg^{-1}$ protein. The specific activity of the purified

 © Cancer Research Campaign 1997

**Figure 3** Immunoblot analysis of purified recombinant NQO1 proteins and NQO1 expression in tumour cells. Immunoblot analysis was performed on purified recombinant NQO1 proteins (0.1 μg) and tumour cell sonicates (25 μg). Reaction conditions are described in Materials and methods. Lane 1, recombinant H460 NQO1 protein; lane 2, H460 cell sonicate; lane 3, H596 cell sonicate; lane 4, recombinant BE NQO1 protein; lane 5, BE cell sonicate protein from cells expressing the coding region from the BE cell line, which contains the homozygous C to T point mutation at position 609, produced a protein with 2% of the specific activity of the wild-type recombinant protein.

### Frequency of the C to T point mutation

To clarify the prevalence of the C to T point mutation at position 609 of the NQO1 cDNA, a PCR–RFLP analysis was used to screen tumour and uninvolved tissue from 45 lung cancer patients ranging in age from 33 to 81 years. Normal tissue samples from these patients included uninvolved lung tissue or blood samples from these individuals. Table 2 shows the results of this screening. A total of 51% of the lung cancer samples were homozygous for the wild-type protein, while 42% were heterozygous for the mutation and 7% were homozygous for the mutation.

Histologically, the tumour tissues examined were composed of squamous cell cancers, adenocarcinomas, small-cell and large-cell lung cancers, bronchioalveolar cancers, a carcinoid tumour and an adenosquamous cancer. Tumour samples homozygous for the mutation included a carcinoid tumour and two adenocarcinomas with bronchioalveolar components. Tumour samples heterozygous for the mutant allele were composed primarily of squamous cell cancers (45%) and adenocarcinomas (40%) with one small-cell lung cancer and one adenosquamous cancer. Tumour samples homozygous for the wild-type allele were composed of 48% squamous cell cancers, 28% adenocarcinomas, 12% small-cell lung cancers, 8% bronchioalveolar cancers and one large-cell lung cancer.

### DISCUSSION

The enzymatic activities of the recombinant proteins presented here show that the purified recombinant NQO1 protein carrying the proline to serine substitution at amino acid 187 has only 2% of the enzymatic activity of the wild-type enzyme. In our previous work, we speculated that the proline to serine substitution in the NQO1 enzyme could adversely affect the pyridine nucleotide-binding site of the enzyme. Subsequently, Ma et al (1992) demonstrated that the glycine residue at position 150, the serine residue at position 151 and the tyrosine residue at position 155 of the NQO1 protein were essential for pyridine nucleotide binding, and that the tyrosine at position 128 was important for dicoumarol binding. This suggests that the pyridine nucleotide-binding site may be unaffected by the loss of the proline residue at position 187. The crystal structure of the rat enzyme published recently by Li et al (1995) has defined the binding sites for both pyridine nucleotide and quinone substrates. This work indicates extensive overlap between these binding sites and confirms that both binding sites are not directly affected by the proline substitution at position 187. The crystal structure indicates that the proline at position 187 is located at the end of a beta sheet and is adjacent to an exposed loop (Li et al, 1995). A mutation at this position may, therefore, cause structural alterations in this area and compromise loop stability. The effects of the proline to serine substitution at position 187 on protein structure are, therefore, unclear at present but obviously have profound consequences on the activity of NQO1. It is important to stress that, although a heterozygous C to T mutation at position 609 may be associated with widely differing NQO1 activities (Kuehl et al, 1995), a homozygous C to T mutation results in a lack of NQO1 protein and activity.

The human NQO1 gene is composed of six exons separated by five introns and is located on chromosome 16. The last exon has four polyadenylation sites, three of which are used, giving rise to 1.2 kb, 1.7 kb and 2.7 kb transcripts (Jaiswal, 1991). PCR analysis showed that the NQO1 gene is transcribed at moderate levels, and our Northern analysis showed transcripts of the correct size in cells that are homozygous or heterozygous for the mutation. This suggests that the NQO1 gene is successfully transcribed and appropriately spliced in cells containing the mutation.

Immunoblot analysis of recombinant and cytosolic NQO1 proteins showed that the recombinant enzyme containing the C to T mutation at position 609 can be expressed in *E. coli*, purified and detected using immunoblots. Our data show that the mutant protein is of the correct molecular weight but almost completely dysfunctional as indicated by activity assays. In addition to the recombinant wild-type protein, the NQO1 enzyme is detectable on immunoblot analysis in the cytosol of the H460 cells, which express the functional NQO1 gene. The mutant enzyme expressed in *E. coli* is also detectable by immunoblot analysis; however, the cytosol from both H596 and BE cells, which carry the homozygous C to T mutation, did not contain any detectable NQO1

**Table 2** Frequency of the C to T point mutation in 45 lung cancer patients stratified by sex and race

| | Tumour tissue | Normal tissue | Male | Female | Caucasian | Hispanic | African–American |
|---|---|---|---|---|---|---|---|
| Total | 45 | 45 | 37 | 8 | 37 | 5 | 3 |
| Wild-type | 23 (51) | 23 (51) | 19 (51) | 4 (50) | 19 (51) | 3 (60) | 1 (33) |
| Heterozygous | 19 (42) | 19 (42) | 17 (46) | 2 (25) | 15 (41) | 2 (40) | 2 (67) |
| Homozygous mutant | 3 ( 7) | 3 ( 7) | 1 ( 3) | 2 (25) | 3 (18) | 0 ( 0) | 0 ( 0) |

Tumour tissue and uninvolved tissue (either lung or blood) was tested from each patient as described in Materials and methods. Numbers indicate individual tissue samples, which were homozygous for the wild-type NQO1 gene, heterozygous for the mutation (heterozygous) or homozygous for the mutation (homozygous mutant). Numbers in parenthesis indicate the percentage of samples in each group.

© Cancer Research Campaign 1997

enzyme using both immunoblot and activity assays. This experiment was performed using both monoclonal and polyclonal antibodies to NQO1. Therefore, although activity studies show the purified mutant proteins have detectable but very low activity, our data suggest that reason for the lack of enzymatic activity in cells homozygous for the C to T point mutation at position 609 is owing to an absence of NQO1 protein.

Recently Rosvold et al (1995*a*) detected the same C to T substitution at position 609 in a Centre d'Etude Polymorphisme Humain (CEPH) reference panel composed of 82 parents and additional family members. The mutant allele appeared in the CEPH panel with a frequency of 0.13 in a manner consistent with Mendelian inheritance. This group tested the association of this mutation with lung cancer, and their preliminary evidence suggested that the mutant allele was over-represented in lung cancer cases. Preliminary data discussed in a recent exchange of letters was conflicting with respect to an increased prevalence of the NQO1 polymorphism in patients with colon cancer (Rosvold et al, 1995*b*; Kolesar et al, 1995). The data we present here show a frequency of 7% of both tumour and normal tissue samples homozygous for the mutation, while 42% of the samples were heterozygous for the mutation. The presence of the mutation in matched lung and blood samples suggests that, in these patients, the mutation is neither tissue nor tumour specific, but represents a true polymorphism. Polymorphisms have been established in numerous phase I and phase II metabolic enzymes (Gonzalez and Idle, 1994). Examples include many members of the cytochrome P450 family of metabolic enzymes, as well as oxidases, reductases and esterases. Polymorphisms in phase II metabolic enzymes include sulpho, acetyl, methyl and glutathione transferases. These polymorphisms are associated with significant alterations in response and susceptibility to xenobiotics (Caporaso et al, 1991). We have shown that NQO1 is important in protection against benzene-derived quinones (Ross et al, 1990; Ganousis et al, 1992). In very recent work, we have demonstrated that workers with the NQO1 polymorphism are at increased risk of benzene-induced decreases in white blood cell count relative to matched controls (Rothman et al, 1996).

A polymorphism leading to a lack of NQO1 activity is of special significance in that the enzyme acts as a protective measure against oxidative damage produced by a wide range of naturally occurring and xenobiotic quinones, which undergo redox cycling subsequent to two-electron reduction by NQO1 (Prochaska et al, 1992; Zhang et al, 1992; Enger et al, 1994). A lack of NQO1 may have implications for both chemoprotection and chemoprevention. Indeed, the potential benefit of chemopreventive agents, which act through the induction of NQO1, could be significantly decreased in populations homozygous for this mutation. Additionally, the presence of this polymorphism presents significant problems in the exploitation of the elevated NQO1 activity of certain tumours by administering anti-tumour quinones, which require bioreductive activation. In an attempt to predict the clinical response of tumours to these drugs, it has been suggested that NQO1 expression in tumour samples could be evaluated by PCR before drug treatment. The presence of this mutation would lead to tumour types that are unresponsive to these drugs, despite apparent elevated NQO1 gene expression as detected by PCR. As such, it will be necessary to confirm the absence of the mutation at position 609 using PCR–RFLP analysis or, more importantly, the presence of NQO1 activity in these samples for studies of this kind to be successful.

In summary, we have characterized a proline to serine mutation in NQO1, which results in a complete loss of enzyme activity. Although purified mutant NQO1 had only 2% of wild-type activity, the lack of enzyme activity appears to be caused by a complete absence of NQO1 protein. The presence of the homozygous mutation in matched lung and blood samples suggests that this mutation represents a true polymorphism and was found to be present in 7% of 45 matched sets of human lung tumours and paired uninvolved tissue. These results may have implications for cancer therapy, chemoprotection and chemoprevention.

### ABBREVIATIONS

NQO1, NAD(P)H:quinone oxidoreductase 1; NSCLC, non-small-cell lung cancer; SCLC, small-cell lung cancer; PCR, polymerase chain reaction; RFLP, restriction fragment length polymorphism.

### ACKNOWLEDGEMENT

This study was supported by RO1 CA 51210 and NCI Spore in Lung Cancer grant CA 58187.

### REFERENCES

Beall HD, Mulcahy RT, Siegel D, Traver RD, Gibson NW and Ross D (1994) Metabolism of bioreductive antitumor compounds by purified rat and human DT-diaphorases. *Cancer Res* **54:** 3196–3201

Benson A, Hunkeler MJ and Talalay P (1980) Increase of NAD(P)H:quinone reductase by dietary antioxidants: possible role in protection against carcinogenesis and toxicity. *Proc Natl Acad Sci USA* **77:** 5216–5220

Bradford M (1976) A rapid and sensitive method for the quantitation of microgram quantities of protein utilizing the principle of protein-dye binding. *Biochemistry* **72:** 248–254

Caporaso N, Landi MT and Vineis P (1991) Relevance of metabolic polymorphisms to human carcinogenesis: evaluation of epidemiologic evidence. *Pharmacogenetics* **1:** 4–19

Edwards YH, Potter J, and Hopkinson DA (1980) Human FAD-dependent NAD(P)H diaphorase. *Biochem J* **187:** 429–436

Eickelmann P, Ebert T, Warskulat U, Schulz WA and Sies H (1994*a*) Expression of NAD(P)H:quinone oxidoreductase and glutathione *S*-transferases alpha and pi in human renal cell carcinoma and in kidney cancer-derived cell lines. *Carcinogenesis* **15:** 219–225

Eickelmann P, Schulz WA, Rohde D, Schmitz-Drager B and Sies H (1994*b*) Loss of heterozygosity at the NAD(P)H: quinone oxidoreductase locus associated with increased resistance against mitomycin C in a human bladder carcinoma cell line. *Biol Chem Hoppe-Seyler* **375:** 439–445

Enger PA, Kensler TW, Prestera T, Talalay P, Libby AH, Joyner HH and Curhey TJ (1994) Regulation of phase 2 enzyme induction by oltipraz and other dithiolethiones. *Carcinogenesis* **15:** 177–181

Ernster L (1967) DT-Diaphorase. *Methods Enzymol* **10:** 309–317

Faeder EJ and Siegel LM (1973) A rapid micromethod for determination of FMN and FAD in mixtures. *Anal Biochem* **53:** 332–336

Ganousis LG, Goon D, Zyglewska T, Wu KK and Ross D (1992) Cell-specific metabolism in mouse bone marrow stroma. Studies of activation and detoxification of benzene metabolites. *Mol Pharmacol* **42:** 531–536

Gibson NW, Hartley JA, Butler J, Siegel D and Ross D (1992) Relationship between DT-diaphorase-mediated metabolism of a series of aziridinylbenzoquinones and DNA damage and cytotoxicity. *Mol Pharmacol* **42:** 531–536

Gibson NW, Phillips RM and Ross D (1994) Mitomycin C. *Cancer Chemother Biol Response Modifiers* **15:** 51–57

Gonzalez FJ and Idle JR (1994) Pharmacogenetic phenotyping and genotyping. Present status and future potential. *Clin Pharmacokinet* **26:** 59–70

Jaiswal AK (1991) Human NAD(P)H:quinone oxidoreductase (NQO1) gene structure and induction by dioxin. *Biochemistry* **30:** 10647–10653

Jaiswal KA, Mcbride OW, Adesnik M and Nebert DW (1988) Human dioxin-inducible cytosolic NAD(P)H:menadione oxidoreductase. cDNA sequence and localization of gene to chromosome 16. *J Biol Chem* **263:** 13572–13578

© Cancer Research Campaign 1997

Kuehl BL, Paterson JWE, Peacock JW, Paterson MC, and Rauth AM (1995) Presence of a heterozygous substitution and its relationship to DT-diaphorase activity. *Br J Cancer* **72:** 555–561

Kolesar JM, Burris HA and Kuhn JG (1995) Re: detection of a point mutation in NQO1 (DT-diaphorase) in a patient with colon cancer. *J Natl Cancer Inst* **87:** 1022–1024

LI R, Bianchet MA, Talalay P and Amzel LM (1995) The three-dimensional structure of NAD(P)H:quinone reductase, a flavoprotein involved in cancer chemoprevention and chemotherapy: mechanism of the two-electron reduction. *Proc Natl Acad Sci USA* **92:** 8846–8850

MA Q, CUI K, Xiao F, LU Ayh and Yang CS (1992) Identification of a glycine-rich sequence as an NAD(P)H-binding site and tyrosine 128 as a dicumarol-binding site in rat liver NAD(P)H:quinone oxidoreductase by site-directed mutagenesis. *J Biol Chem* **267:** 22298–22304

Malkinson AM, Siegel D, Forrest GL, Gazdar AF, Oie HK, Chan DC, Bunn PA, Mabry M, Dykes DJ, Harrison SD and Ross D (1992) Elevated DT-diaphorase activity and messenger RNA content in human non-small cell lung carcinoma: relationship to the response of lung tumor xenografts to mitomycin Cl. *Cancer Res* **52:** 4752–4757

Marshall RS, Paterson MC and Rauth AM (1991*a*) DT-diaphorase activity and mitomycin C sensitivity in non-transformed cell strains derived from members of a cancer-prone family. *Carcinogenesis* **12:** 1175–1180

Marshall RS, Paterson MC and Rauth AM (1991*b*) Studies on the mechanism of resistance to mitomycin C and porfiromycin in a human cell strain derived from a cancer-prone individual. *Biochem Pharmacol* **41:** 1351–1360

Peppel K and Baglioni C (1990) A simple and fast method to extract RNA from tissue culture cells. *Biotechniques* **9:** 711–713

Phillips RM, Sarang M and Gibson NW (1993) Semi-quantitative measurement of gene expression byRT–PCR: a cautionary tale. *Int J Oncol* **3:** 1097–1102

Prochaska HJ, Santamaria AB and Talalay P (1992) Rapid detection of inducers of enzymes that protect against carcinogens. *Proc Natl Acad Sci USA* **89:** 2394–2398

Ross D, Siegel D, Gibson NW, Pacheco D, Thomas DJ, Reasor M and Wierda D (1990) Activation and deactivation of quinones catalyzed by DT-diaphorase. Evidence for bioreductive activation of diaziquone (AZQ) in human tumor cells and detoxification of benzene metabolites in bone marrow stroma. *Free Rad Res Commun* **8:** 373–381

Ross D, Siegel D, Beall HD, Prakash AS, Mulcahy RT and Gibson NW (1993) DT-diaphorase in activation and detoxification of quinones. Bioreductive activation of mitomycin C. *Cancer Metast Rev* **12:** 83–101

Ross D, Traver RD, Siegel D, Kuehl BL, Misra V and Rauth AM (1996) A polymorphism in NAD(P)H: quinone oxidoreductase (NQO1): relationship of a homozygous mutation at position 609 of the NQO1 cDNA to NQO1 activity. *Br J Cancer* **74:** 995–996

Rosvold EA, Mcglynn KA, Lustbader ED and Buetow KH (1995*a*) Identification of an NAD(P)H:quinone oxidoreductase polymorphism and its association with lung cancer and smoking. *Pharmacogenetics* **5:** 199–206

Rosvold EA, Mcglynn KA, Lustbader ED and Buetow KH (1995*b*) Re: detection of a point mutation in NQO1 (DT-diaphorase) in a patient with colon cancer. *J Natl Cancer Ins* **87:** 1022–1024

Rothman N, Traver RD, Smith MT, Hayes RB, LI GL, Campleman S, Dosemecci M, Zhang L, Linet M, Wacholder S, Yin SN and Ross D (1996) Lack of NAD(P)H:quinone oridoreductase activity (NQO1) is associated with increased risk of benzene hematotoxicity. *Proc Am Assoc Cancer Res* **37:** 258

Schlager JJ and Powis G (1990) Cytosolic NAD(P)H:(quinone-acceptor) oxidoreductase in human normal and tumor tissue: effects of cigarette smoking and alcohol. *Int J Cancer* **45:** 403–409

Siegel D, Gibson NW, Preusch, PC and Ross D (1990) Metabolism of mitomycin C by DT-diaphorase: role in mitomycin C-induced DNA damage and cytotoxicity in human colon carcinoma cells. *Cancer Res* **50:** 7483–7489

Sharkis DH and Swenson RP (1989) Purification by cibacron blue F3GA dye affinity chromatography and comparison of NAD(P)H:quinone reductase (E.C.1.6.99.2) from rat liver cytosol and microsomes. *Biochem Biophys Res Commun* **161:** 434–441

Traver RD, Horikoshi T, Danenberg KD, Stadlbauer THW, Danenberg PV, Ross D and Gibson NW (1992) NAD(P)H:quinone oxidoreductase gene expression in human colon carcinoma cells: characterization of a mutation which modulates DT-diaphorase activity and mitomycin sensitivity. *Cancer Res* **52:** 797–802

Traver RD, Phillips RM, Gibson NW and Ross D (1995) A point mutation in both human lung and colon carcinoma cell lines leading to a loss of DT-diaphorase activity. *Proc Am Assoc Cancer Res* **36:** 525

Walton MI and Workman P (1990) Enzymology of the reductive bioactivation of SR 4233. A novel benzotriazine di-N-oxide hypoxic cell cytotoxin. *Biochem Pharmacol* **39:** 1735–1742

Wattenberg LW (1985) Chemoprevention of cancer. *Cancer Res* **45:** 1–8

Zhang Y, Talalay P, Cho CG and Posner GH (1992) A major inducer of anticarcinogenic protective enzymes from broccoli: isolation and elucidation of structure. *Proc Natl Acad Sci USA* **89:** 2399–2403

© Cancer Research Campaign 1997